# Renal‑rotation techniques in retroperitoneoscopic adrenalectomy for giant pheochromocytomas: a clinical intervention study with historical controls

**DOI:** 10.1186/s12894-023-01221-w

**Published:** 2023-03-29

**Authors:** Ruizhi Xue, Caoyang Hu, Zhongyi Zheng, Liang Wei, Xiaobin Yuan, Xiao Lyu, Pengliang Shen, Jun Li, Xiaoming Cao

**Affiliations:** Urology Department, The First Hospital of Shanxi Medical University, Jiefang South Road, Taiyuan, Shanxi 030000 China

**Keywords:** Adrenalectomy, Giant pheochromocytoma, Clinical intervention, Comparative study

## Abstract

**Background:**

Dealing with the giant pheochromocytomas (maximum diameter ≥ 6 cm) has long been a tough challenge for urologists. We introduced a new retroperitoneoscopic adrenalectomy method modified with renal-rotation techniques to treat giant pheochromocytomas.

**Methods:**

28 diagnosed patients were prospectively recruited as the intervention group. Meanwhile, by referring to the historical records in our database, matched patients who had undergone routine retroperitoneoscopic adrenalectomy (RA), transperitoneal laparoscopic adrenalectomy (TA), or open adrenalectomy (OA) for giant pheochromocytomas were selected as controls. Perioperative and follow-up data were collected for comparative assessment.

**Results:**

Among all the groups, the intervention group had the minimal bleeding volume (28.93 ± 25.94 ml, p < 0.05), the least intraoperative blood pressure variation (59.11 ± 25.68 mmHg, p < 0.05), the shortest operation time (115.32 ± 30.69 min, p < 0.05), the lowest postoperative ICU admission rates (7.14%, p < 0.05), and shortest drainage time length (2.57 ± 0.50 days, p < 0.05). Besides, compared with TA and OA groups, intervention group was also characterized by lower pain scores (3.21 ± 0.63, p < 0.05), less postoperative complications (p < 0.05), earlier diet initiation time (1.32 ± 0.48 postoperative days, p < 0.05) and ambulation time (2.68 ± 0.48 postoperative days, p < 0.05). Follow-up blood pressure and metanephrine and normetanephrine levels in all intervention group patients remained normal.

**Conclusion:**

Compared with RA, TA, and OA, retroperitoneoscopic adrenalectomy with renal-rotation techniques is a more feasible, efficient, and secure surgical treatment for giant pheochromocytomas.

**Trial registration:**

This study has been prospectively registered on the Chinese Clinical Trial Registry website (ChiCTR2200059953, date of first registration: 14/05/2022).

**Supplementary Information:**

The online version contains supplementary material available at 10.1186/s12894-023-01221-w.

## Background

Pheochromocytomas are catecholamine-producing neuroendocrine tumors derived from the adrenal neuroectoderm. The estimated incidence of pheochromocytomas ranges from 0.005 to 0.1% of the general population and from 0.1 to 0.2% of the adult hypertensive population [1]. Although typical symptoms of pheochromocytomas are characterized by the triad of headaches, palpitations, and profuse sweating, up to 20% of the patients have no obvious clinical symptoms [2]. These “silent” pheochromocytomas can remain unnoticed for years and grow into giant sizes at the time of diagnosis [3, 4].

To date, retroperitoneoscopic adrenalectomy (RA), transperitoneal laparoscopic adrenalectomy (TA), and open adrenalectomy (OA) are frequently used techniques for removing adrenal tumors including pheochromocytoma [5, 6]. However, resection of giant pheochromocytomas (GP), which are commonly defined by a maximum diameter ≥ 6 cm, has always been a major challenge for such surgeries [7–9]. The limited surgical space and inadequate exposure of the lesion can cause extreme difficulties during tumor manipulation, and also brings high risks of hemorrhage and catecholamine crisis. In this study, our team developed a surgical method that applied renal‑rotation techniques in RA. Through our comparative assessment, we found that our renal-rotation techniques can effectively increase the surgical space and enhance the safety of adrenalectomy for GP.

## Methods

Considering the relative rarity of GP, we adopted a historical control study design (the interventional arm was established by prospectively recruiting newly diagnosed patients, while matched historical cases were set as the control arm). The study was approved by the Ethics Committee of the First Hospital of Shanxi Medical University (202206722) and prospectively registered on the Chinese Clinical Trial Registry website (ChiCTR2200059953, date of first registration: 14/05/2022).

### Inclusion and exclusion criteria

For all the enrolled GP cases, the general inclusion criteria were: age 20 to 60 years; clear preoperative diagnosis of pheochromocytoma; preoperative imaging examination (CT or MRI) showing that the maximum diameter of the tumor was ≥ 6 cm, preoperative volume expansion was completed, and signed the informed consent form.

The exclusion criteria were as follows: age < 20 or > 60 or severely disabled; bilateral pheochromocytomas or ectopic pheochromocytomas; previous history of retroperitoneal infection or surgical operation; severe underlying diseases such as poor control of diabetes, arrhythmia, heart failure, etc.; and refusal to sign the informed consent form.

### Grouping methods

**Fig. 1:**
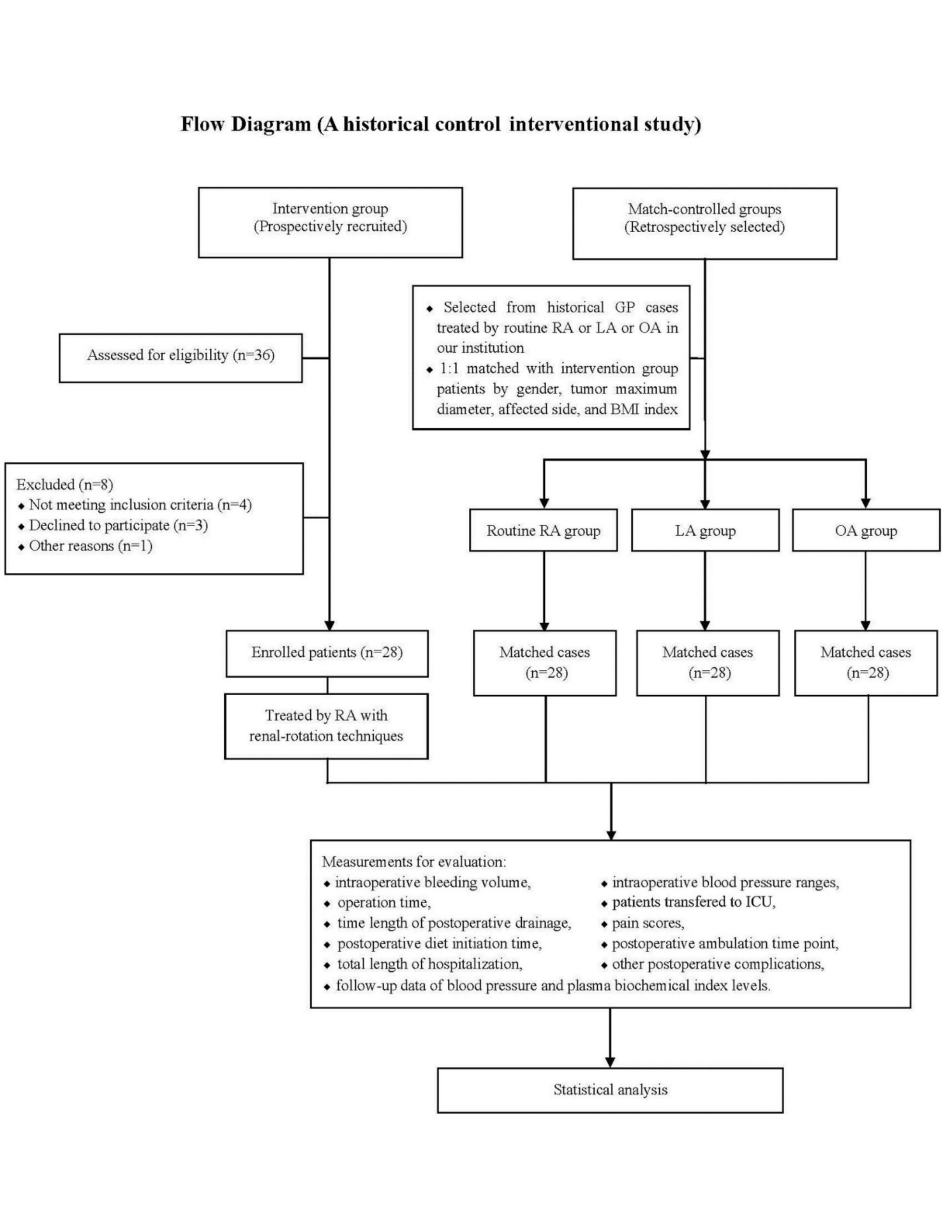
Flow diagram of this clinical interventional study

The intervention group (receiving RA with renal-rotation techniques) was established by prospectively recruiting GP patients, and all the surgeries were performed by two senior surgeons in our department. For the controls, three groups were established by retrospectively enrolling historic GP cases with surgical records of routine RA (without renal-rotation techniques), TA, and OA surgeries which all conducted by the same two surgeons at our institution. To further control the effects of baseline variables, the sex distribution, maximum tumor diameter, affected side, and BMI index of all the controlled cases were matched with the intervention group patients. The enrollment and grouping designs are shown in the flow diagram of Fig. 1.

### Preoperative preparation

Volume extension therapy with alpha-blockers (terazosin, 1–2 mg, tid) was given for 4–8 weeks. A beta blocker (metoprolol) was administered if the patient had tachycardia. The preoperative blood pressure should be stabilized at approximately 120/80 mmHg and the heart rate should be lower than 100 bpm.

### Surgical methods

#### Intervention group (RA with renal‑rotation techniques)

**Fig. 2:**
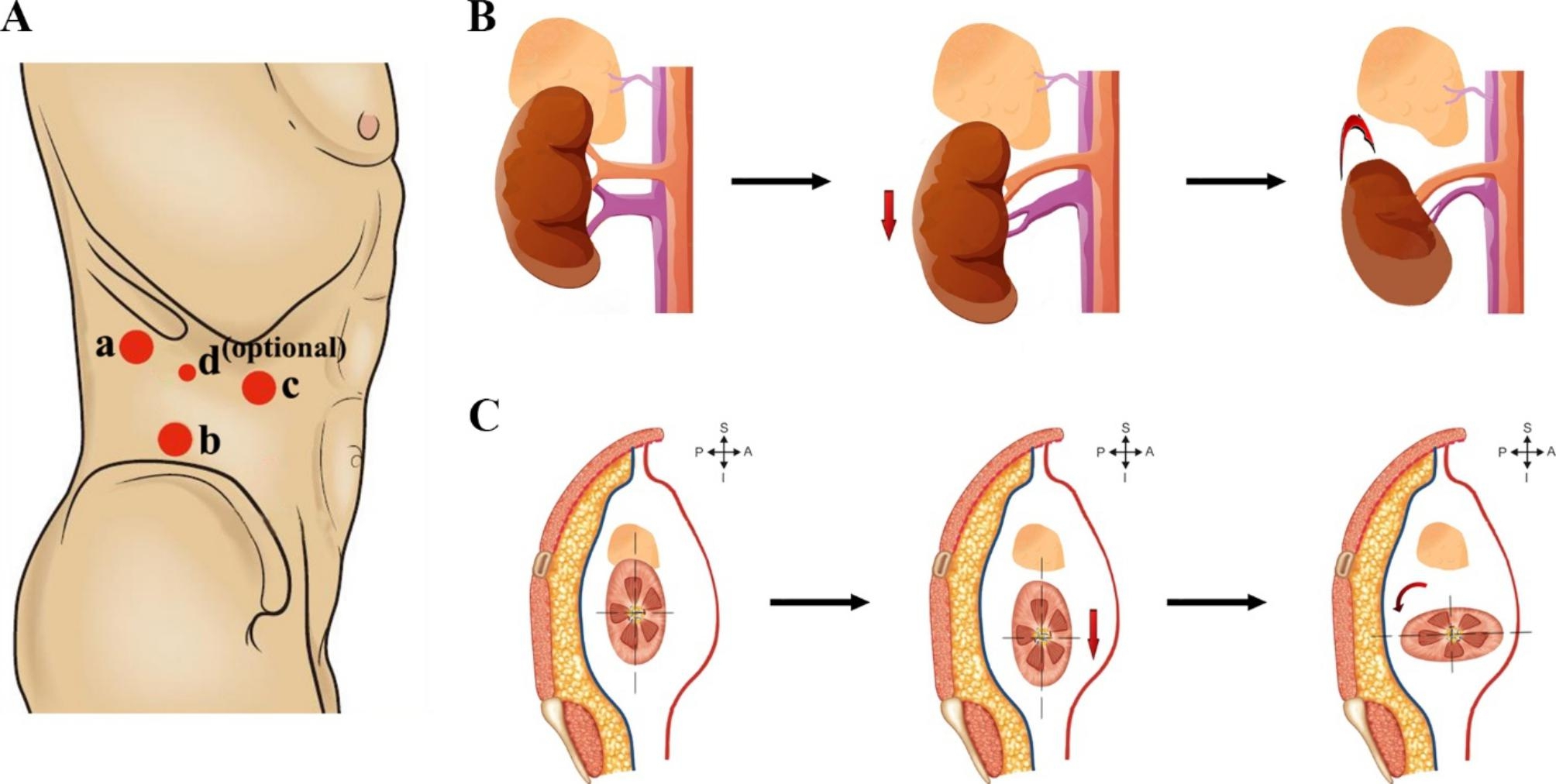
Diagrammatic sketch of ports location and renal-rotation strategy (A) A 2-cm incision is made between the tip of the 12th rib and the iliac crest, then a 12 mm port is placed in the incised site (a); The second 12 mm port is placed 2 cm above the anterior superior iliac spine (b); The third 12 mm port is placed 3 to 4 cm medial and slightly inferior to the first port in the anterior axillary line (c); An alternate site (d) for 5 mm port can be added in the midpoint between a and c sites. (B) The renal rotation processes in the front view of coronal plane. (C) The renal rotation processes in the lateral view of sagittal plane

The patients were placed in the full-flank lateral decubitus position and intubated by the anesthesia team. The surgeon stood facing the patient’s back. The retroperitoneal space was set up by finger dissection, and a three-port (or four-port) channel was established, as shown in Fig. 2A (the general process of renal rotation is sketched in Fig. 2B-C). The CO2 insufflation pressure was set between 12 and 14 mmHg (Same pressure range was applied in the control groups with laparoscopic procedures).

**Fig. 3:**
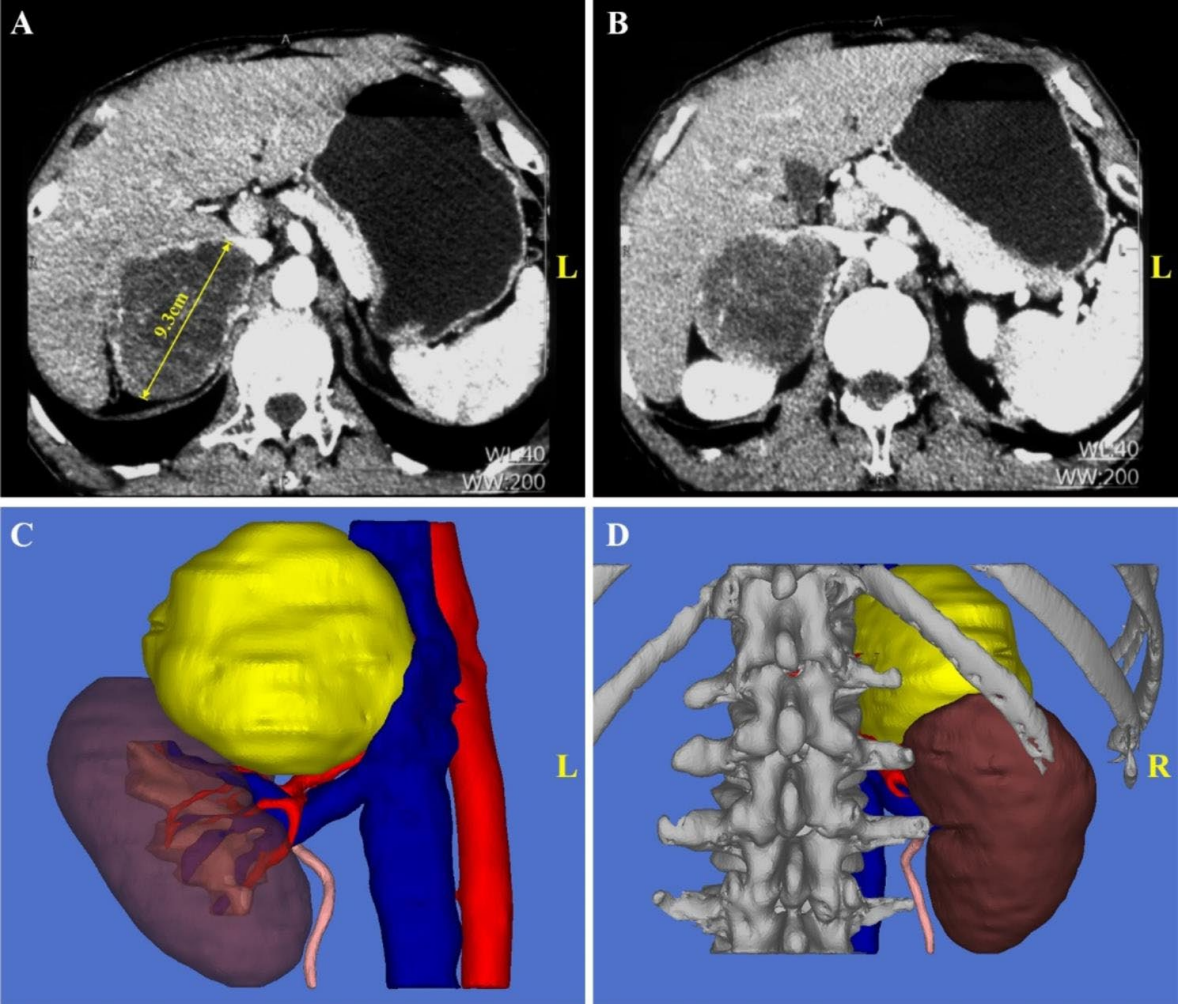
Preoperative imaging data of a patient with typical right-sided giant pheochromocytoma (A) The maximum diameter of the tumor is 9.3 cm. (B) The right kidney is squeezed by the tumor and displaced. (C) The front view of the 3D anatomical location of the tumor. (D) The rear view of the 3D anatomical location of the tumor

**Fig. 4:**
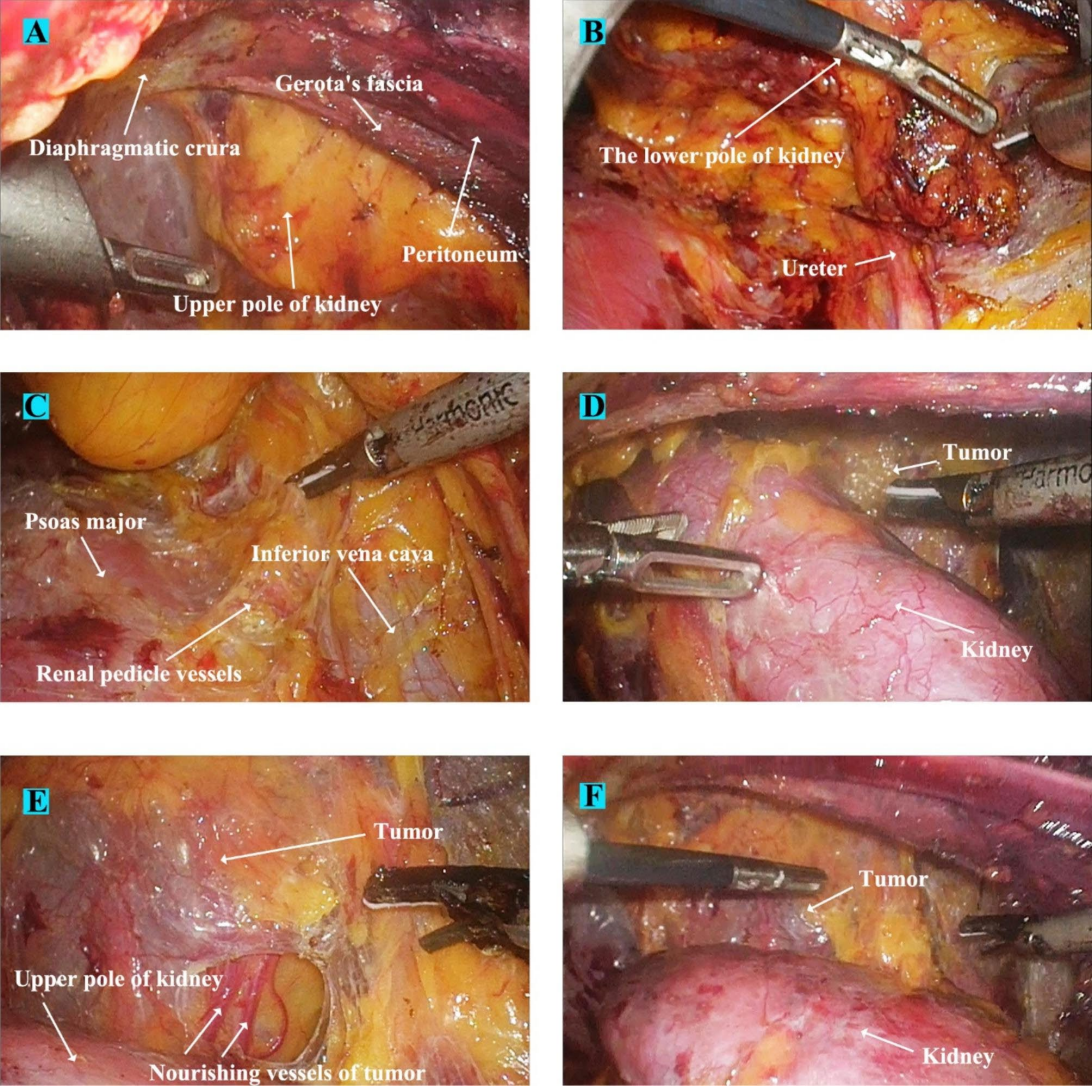
Renal mobilization and tumor exposure surgical processes of a patient with typical right-sided giant pheochromocytoma (A) Gerota’s fascia was adequately incised, incision upper bound reached the diaphragmatic crura. (B) Incision lower bound was down to the middle ureter level, to fully expose the entire kidney and lesion area. (C) Dorsal side of kidney was dissociated along the psoas major fascia, the renal pedicle area and inferior vena cava were carefully exposed. (D) Ventral side of kidney was subsequently dissected, and tumor was progressively exposed. (E) Dragging down the kidney while dissecting its upper pole, then sealing the exposed nourishing vessels in the tumor surface with harmonic scalpel. (F) As the dissection progressed, the kidney had automatically tilted under the influence of gravity

For right-sided GP (exemplified in Fig. 3), after entering the retroperitoneum, the fatty tissue along the peritoneum and Gerota’s fascia were thoroughly removed. Gerota’s fascia was then adequately incised from the upper roof of the retroperitoneal cavity (near the diaphragmatic crura) to the middle ureter level to fully expose the entire kidney and the lesion area (Fig. 4A-B). Next, the renal pedicle area was exposed by dissociating along the psoas major fascia (Fig. 4C). The kidney was thoroughly dissociated and mobilized from the fat layer using a harmonic scalpel along the outer edge (Fig. 4D). During this procedure, caution should be exercised while separating the upper and lower poles of the kidney to prevent injury to the tumor blood vessels, ureter, and inferior vena cava.

**Fig. 5:**
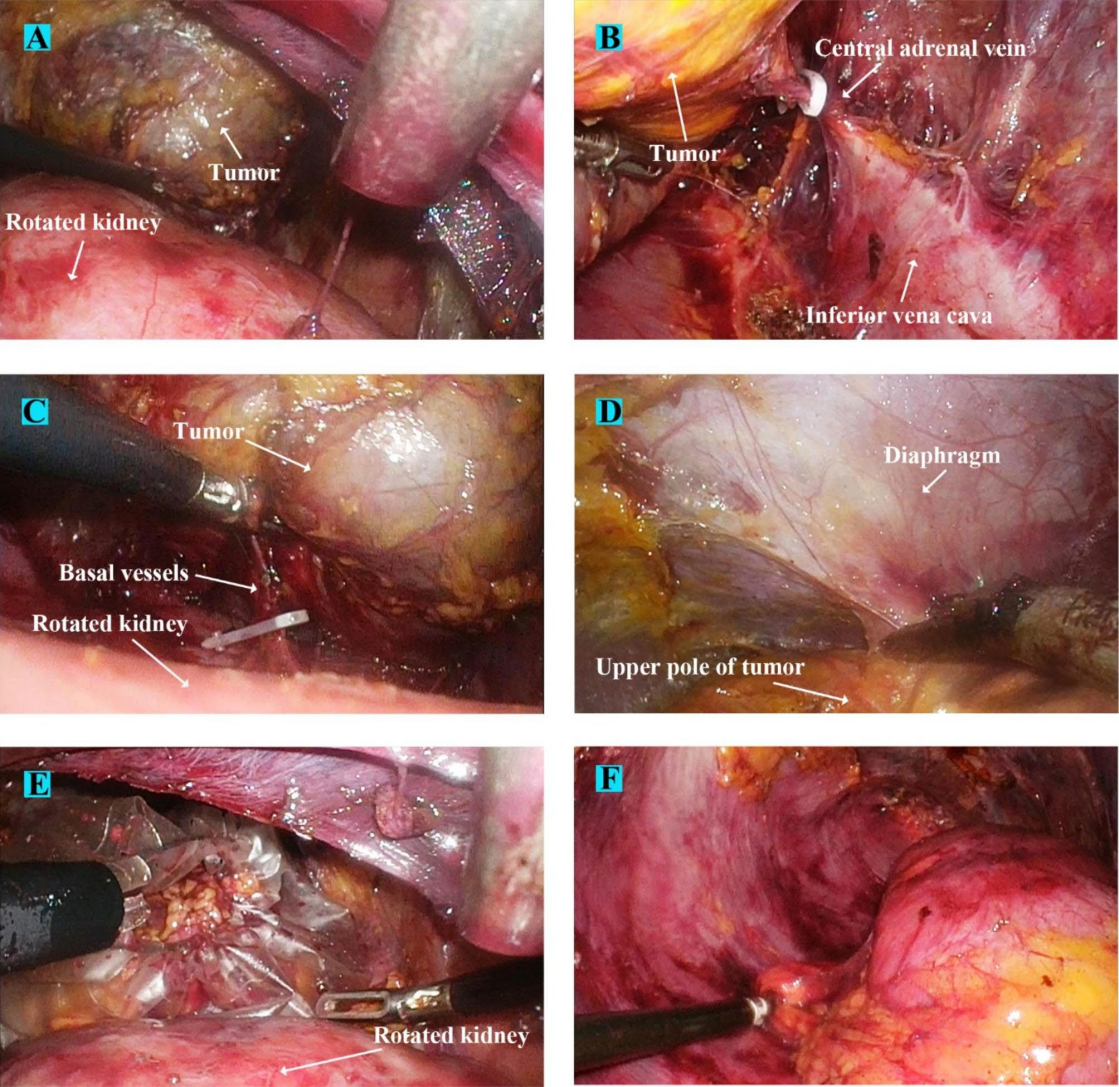
Renal rotation and tumor resection surgical processes of a patient with typical right-sided giant pheochromocytoma (A) Kidney was laid horizontally in the direction of its inclination to provide ample space and excellent field view for tumor resection. (B) The fibrous tissue was dissected along the vena cava to expose the central adrenal vein which was then controlled by Hem-O-Loks. (C) Neoplastic vasa vasorum usually crisscrossed in the base of tumor, and renal rotation offered sufficient space for exposing and sealing these basal vessels. (D) After the thorough dissociation of tumor’s basal part and two flanks, upper pole of tumor was finally dissected. (E) After the tumor was completely freed, it was placed in a plastic retrieval bag and extracted. (F) Finally, the kidney was rotated back to its original position and the surgery was finished

Rotation of the kidney was then conducted in two steps. First, the kidney was dragged downward as far as possible, causing the upper pole to descend adequately (Fig. 4E). In the second step, ventral or dorsal rotation was performed (either direction was decided for better exposure of the tumor) to place the kidney in a horizontal position (Figs. 4F and 5A). Renal blood vessels were closely monitored during the procedure to avoid vascular tears. A fourth port channel can be added to facilitate and maintain the rotations when necessary. After exposing the lesion, we continued dissection medially to identify the vena cava. Fibrous tissue was dissected along the right side of the vena cava to expose and control the right adrenal vein using Hem-O-Loks (Fig. 5B). The tumor was dissected in the following order: the basal part of the tumor, two flanks, and the upper pole of the tumor (Fig. 5C-D). Such an operation sequence could maintain the tumor in a suspended state, which prevented the tumor from flipping down and reduced excessive manipulation during tumor dissection.

**Fig. 6:**
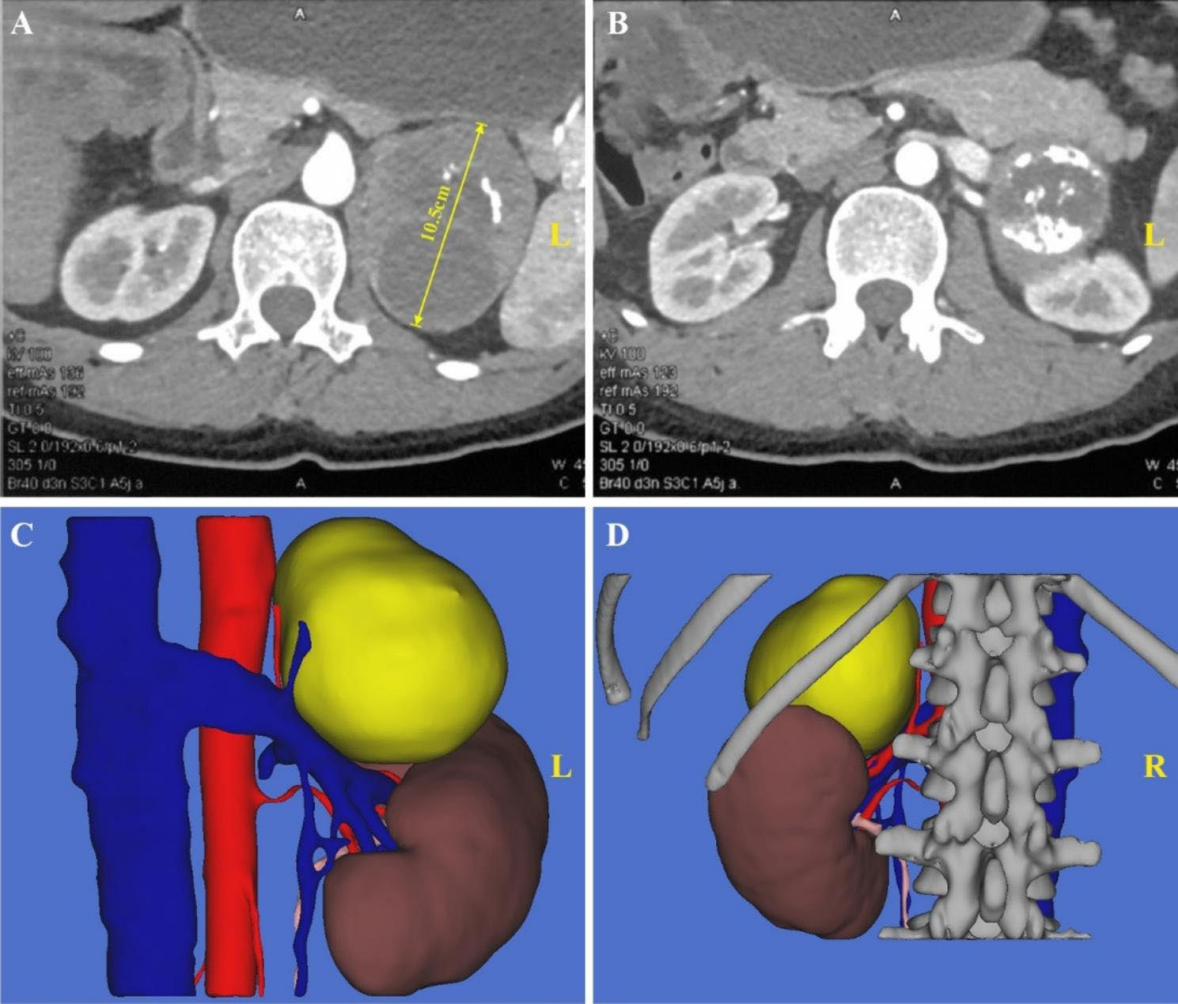
Preoperative imaging data of a patient with typical left-sided giant pheochromocytoma (A) The maximum diameter of the tumor is 10.5 cm. (B) The left kidney is squeezed by the tumor and displaced. (C) The front view of the 3D anatomical location of the tumor. (D) The rear view of the 3D anatomical location of the tumor

**Fig. 7:**
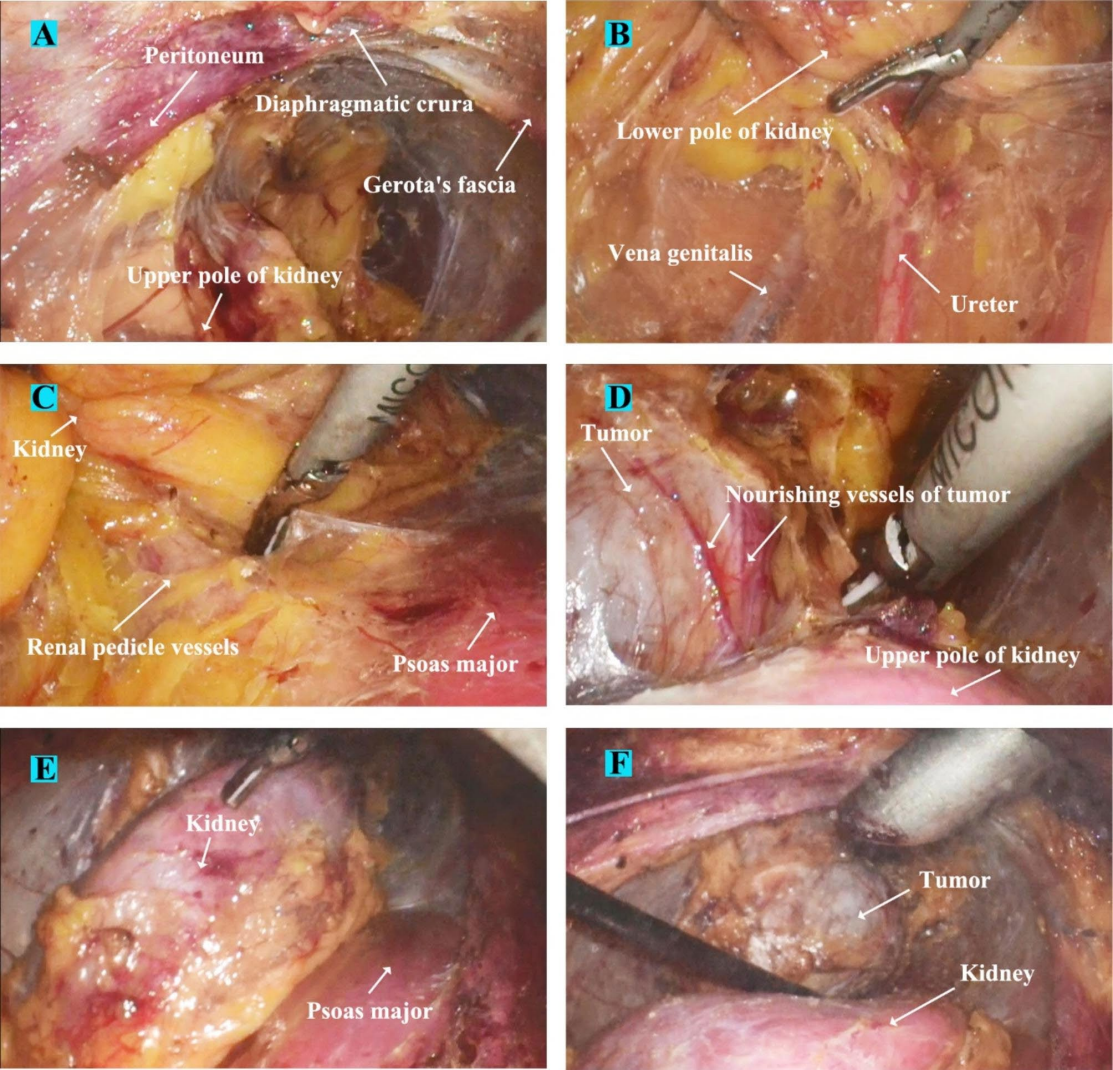
Renal mobilization and tumor exposure surgical processes of a patient with typical left-sided giant pheochromocytoma (A) Gerota’s fascia was adequately incised, incision upper bound reached the diaphragmatic crura. (B) Incision lower bound was down to the middle ureter level, to fully expose the entire kidney and lesion area. (C) Dorsal side of kidney was dissociated along the psoas major fascia, the renal pedicle area was carefully exposed. (D) Dragging down the kidney while dissecting its upper pole, then sealing the exposed nourishing vessels in the tumor surface with harmonic scalpel. (E) The kidney was fully mobilized by circumferentially dissecting its each side. (F) As the dissection progressed, the kidney had automatically tilted under the influence of gravity, and the tumor was progressively exposed

**Fig. 8:**
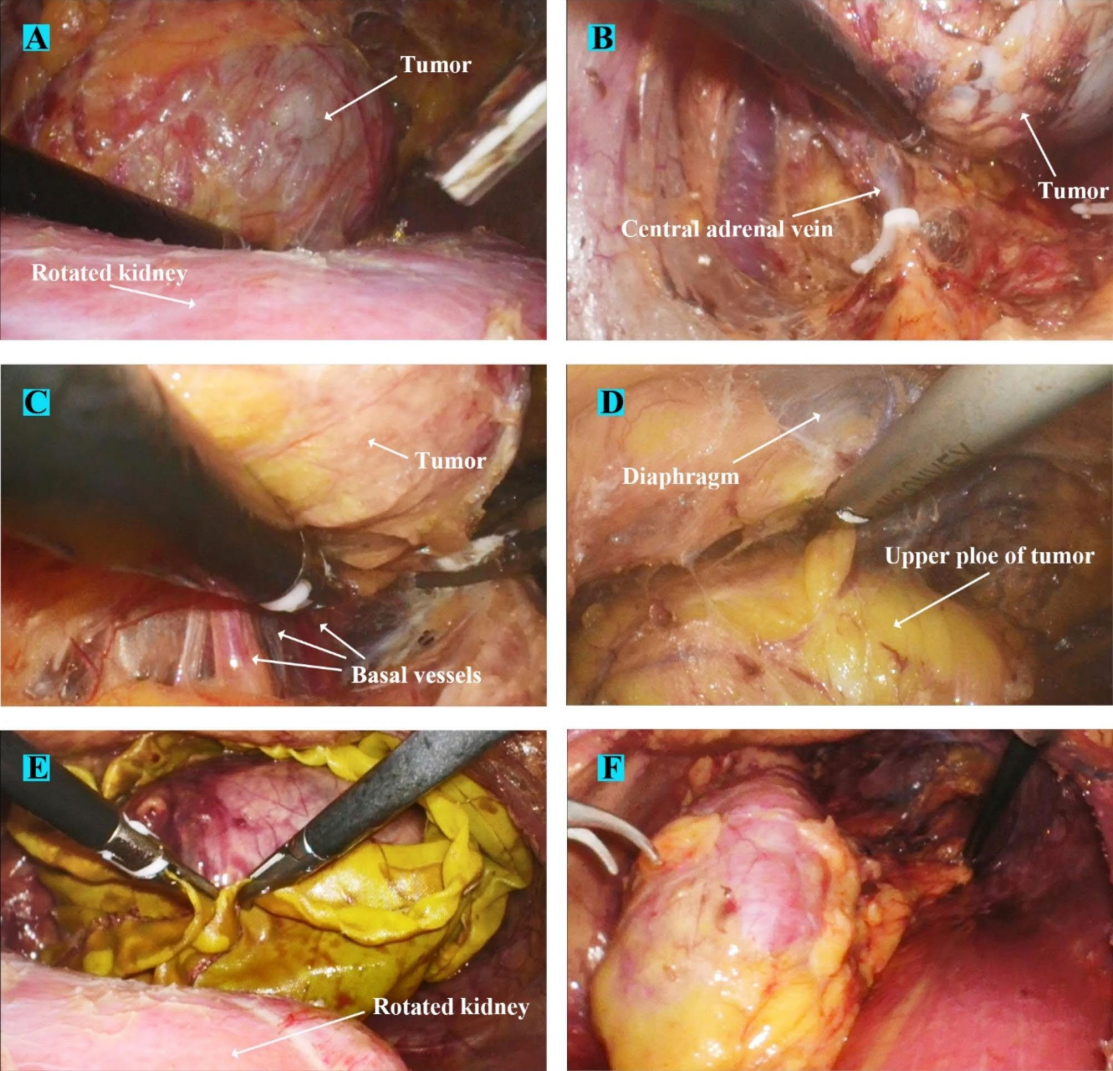
Renal rotation and tumor resection surgical processes of a patient with typical left-sided giant pheochromocytoma (A) Kidney was laid horizontally in the direction of its inclination to provide ample space and excellent field view for tumor resection. (B) The fibrous tissue was dissected along the renal vein to expose the central adrenal vein which was then controlled by Hem-O-Loks. (C) Neoplastic vasa vasorum usually crisscrossed in the base of tumor, and renal rotation offered sufficient space for exposing and sealing these basal vessels. (D) After the thorough dissociation of tumor’s basal part and two flanks, upper pole of tumor was finally dissected. (E) After the tumor was completely freed, it was placed in a retrieval bag and extracted. (F) Finally, the kidney was rotated back to its original position and the surgery was finished

For the left-sided GP ( Fig. 6), the initial dissection and renal rotation processes were the same as those for the right-sided GP (Fig. 7). The main difference was in the adrenal vein-handling phase. After complete exposure of the lesion, the left renal vein was carefully dissected. The fat tissue around the left adrenal vein area at the inferomedial aspect of the adrenal gland was methodically removed, and the central adrenal vein was exposed and controlled by Hem-O-Loks (Fig. 8A-B). Subsequently, tumor dissection was performed similar to the right-sided GP (Fig. 8C-D).

After complete dissection of the tumor, the tumor body was removed (Figs. 5E and 8E). Finally, the kidney was rotated back and fixed by anchoring of some perinephric fat to a lateral attachment to prevent postoperative renal malrotation or nephroptosis (Figs. 5F and 8F), a retroperitoneal drainage tube was placed in place, the trocars were removed, and the wound was closed.

Corresponding surgical videos of right-sided and left-sided GP can be seen on *MEDtube* website with following links: https://medtube.net/urology/medical-videos/34909-renal-rotation-techniques-in-retroperitoneoscopic-adrenalectomy-for-giant-pheochromocytomas-right-sided and https://medtube.net/urology/medical-videos/34910-renal-rotation-techniques-in-retroperitoneoscopic-adrenalectomy-for-giant-pheochromocytomas-left-sided.

#### Routine RA control group

The port placement and initial dissection procedures were the same as those in the intervention group. The main operational differences include the following: (1) the incision length of Gerota’s fascia was shorter and the lower pole of the kidney was not exposed; (2) full kidney mobilization and renal rotation maneuvers were not performed; (3) dissection of the tumor began at its superior border (because this allows the tumor to retract en bloc with the kidney), [10] instead of following the same operation sequence as the intervention group.

#### TA control group

The patient was positioned in a lateral position on a bean bag to open the angle between the ribs and the iliac crest. A three-port channel was used as the working triangulation unit with a camera in the center. The colon was mobilized by cutting its ligament. For right-sided GP, the right triangular ligament of the liver and posterior peritoneum were incised to fully expose the tumor and vena cava. The vena cava was carefully dissected to expose the adrenal vein and was controlled using a Hem-O-Lok. For left-sided GP, the lateral splenic ligaments were divided and the adrenal vein was gently controlled by dissection along the exposed left renal vein. Subsequently, the tumor was disassociated circumferentially, and nourishing vessels were sealed.

#### OA control group

A transverse subcostal or chevron incision was made to ensure excellent exposure. The fascia, rectus sheath, and lateral abdominal musculature were progressively divided to allow entry into the retroperitoneal cavity. If the operating space was still limited, the peritoneum was further incised. Adrenal vein control and tumor dissection procedures were similar to those in the TA group.

### Measurements for evaluation

The following perioperative measurement data were documented for comparative evaluation: intraoperative bleeding volume, intraoperative blood pressure variation (maximum range), operation time, duration of ICU stay, length of postoperative drainage, pain scores, postoperative diet initiation time, postoperative ambulation time point, total length of hospitalization, and other postoperative complications.

One month after discharge, the patients were followed up to acquire self-monitoring blood pressure data. Plasma metanephrine and normetanephrine levels were rechecked routinely.

### Statistical analysis

Continuous variable data were expressed as mean ± standard deviation (SD), and compared values were analyzed using the independent t-test. Non-normally distributed continuous data, which were analyzed using the Mann-Whitney U test, are presented as medians (interquartile range [IQR], 25-75%). Categorical data were expressed as numbers and percentages and analyzed using Pearson’s chi-square test with continuity correction or Fisher’s exact probability method. All statistical calculations were performed using SPSS (Statistical Product and Service Solution) version 10.0 (IBM, Chicago, Illinois, US), and *p* values less than 0.05 were considered statistically significant for all data.

## Results

Details of the patients’ preoperative baseline characteristics are shown in Supplementary Table 1 (Supplementary Material). All surgical treatments in this study were performed by the same two senior surgeons from our department, and there were no intraoperative unplanned modifications of the surgical approaches. As shown in Supplementary Table 2 (Supplementary Material), intraoperative data statistics revealed that intervention group patients had the minimal bleeding volume (Mean ± SD:28.93 ± 25.94 ml; Median [IQR]:20.00 [10.00–50.00], *p* < 0.05), the shortest operation time (115.32 ± 30.69 min, *p* < 0.05), and the least systolic pressure ranges (59.11 ± 25.68 mmHg, *p* < 0.05). For postoperative measurements, the postoperative ICU admission rates (7.14%, *p* < 0.05) and postoperative drainage time length (2.57 ± 0.50 days, *p* < 0.05) were the lowest in the intervention group. Besides, compared with OA and TA groups, intervention group patients were also characterized by a faster convalescence process with advantages of earlier diet initiation time (1.32 ± 0.48 days after surgery, *p* < 0.05) and ambulation time points (2.68 ± 0.48 days after surgery, *p* < 0.05). As for the pain score evaluation, except for the OA group (7.04 ± 0.64, *p* < 0.05), the rest showed no significant differences.

Postoperative gastrointestinal complaints (including abdominal distention, flatulence, and belching) were the most common postoperative complications, occurring most frequently in the TA (57.14%) and OA (32.14%) groups. Two patients in the OA group exhibited delayed wound healing. The incidence rates of other complications, such as hypotension, hypoglycemia, and deep venous thrombosis (DVT), showed no significant difference between the groups. Follow-up data revealed that one patient in the OA group had poor blood pressure control and metanephrine elevation (elevated 6-fold), and he was later diagnosed with metastases after resection of the primary tumor. The pathological results, blood pressure ,metanephrine and normetanephrine levels of all remaining patients remained normal. CT rechecking result showed no report of postoperative renal malrotation or nephroptosis of all the patients.

## Discussion

Resection of giant pheochromocytomas has long been regarded a difficult challenge for surgeons. Surgical difficulties arise from not only the narrow operating space caused by the tumor occupation, but also the risks of hemorrhage and hazardous blood pressure elevation with fatal cardiac arrhythmias due to tumor overgrowth and exuberant synthesis of catecholamine. For these reasons, minimally invasive adrenalectomy surgeries in nowadays still have many intractable difficulties in handling giant pheochromocytomas [7–9]. To improve this difficult situation, we have learned from the renal-rotation techniques in retroperitoneal partial nephrectomy, [11, 12] and further modified and applied it in the treatment of giant pheochromocytoma.

With the help of renal-rotation techniques, we have successfully increased the operating space by fully mobilizing and rotating the kidney downwards, making it feasible to extensively expose and safely resect GP. The key points of our methods lie in two steps, one is fully mobilizing the kidney, and the other is maintaining the suspended position of the adrenal tumor (the superior border of the tumor is dissected last). After thorough renal mobilization and rotation, the blocking effect from the kidney can be removed to the maximum extent. Besides, downward dragging and rotation of the kidney can provide excellent field exposure of the tumor bottom, which greatly facilitates dissection of the tumor inferior margin and sealing of nourishing vessels on the tumor surface (Figs. 4E and 7D). Following full rotation of the kidney, the tumor will usually subsequently hang down, and it is important to maintain the suspended position of the tumor. A suspended tumor can make it easy to adjust the angle, which can not only avoid excessive direct manipulation of the tumor but also facilitate exposure of the basal vascular network (Figs. 5A-C and 8A-C). The results of our comparative assessments between the routine RA group and intervention group demonstrated that the renal-rotation techniques significantly reduced the operation time (once we finished the resection of GP in just 44 min), blood loss, and intraoperative blood pressure variation. Such outcomes indicate that our innovative surgical techniques can effectively ease the surgical difficulties associated with RA and enhance patient safety during GP adrenalectomy.

In clinical practice, many surgeons have alternatively chosen TA and OA for patients with GP to acquire adequate operative space and guarantee safety. Therefore, we also included these two surgical approaches in our comparative assessments. The results showed that our intervention group still had advantages in intraoperative measurements as well as postoperative complications and recovery. Among the reported complications, postoperative gastrointestinal complaints were the most common and were mostly contributed by the OA and TA groups. Based on our experience and relevant literature, this is because the gastrointestinal tract is inevitably disturbed when the transabdominal approach is adopted [13, 14].

Although our renal rotation techniques can effectively increase the operating space for GP resection, it must be noted that there is still upper limit for this method. In our experience, RA with renal rotation could barely handle large tumors with diameters > 12 cm, it is mainly recommended for GP with diameters ranging from 6 to 12 cm. However, considering the rarity of reported GP cases that were beyond the upper range limit, we believe that our method is suitable for most patients with GP. Also, it should be noted that this is a single-center study, and all the described improvements were summarized by comparing against our own previous surgical protocols. The efficiency and effects of our techniques may be inconsistent in other countries/hospitals, due to the regional differences of surgical standards. In addition, this study did not compare the effectiveness of our method with robot-assisted adrenalectomy, which has been gradually popularized for GP treatment owing to its excellent visualization and fine manipulation. We believe that our method is more economical and has edges in learning curve as well as accessibility. Nevertheless, a concrete comparative study is required in the future to fully clarify their advantages and disadvantages.

## Conclusions

Compared with routine retroperitoneoscopic adrenalectomy, transperitoneal laparoscopic adrenalectomy, and open adrenalectomy, retroperitoneoscopic adrenalectomy with renal-rotation techniques is a more feasible, efficient, and secure surgical treatment for giant pheochromocytomas.

## Electronic supplementary material

Below is the link to the electronic supplementary material.

Supplementary Material 1: Tables of perioperative and follow-up data

## Data Availability

The datasets used and/or analysed during the current study are available from the corresponding author on reasonable request.
