# Supplementary material for: Renal‑rotation techniques in retroperitoneoscopic adrenalectomy for giant pheochromocytomas: a clinical intervention study with historical controls
Source: BMC Urol. 2023 Mar 29;23:47. doi: 10.1186/s12894-023-01221-w (PMC10061753; doi:10.1186/s12894-023-01221-w)
Supplement: Supplementary file 1 — Supplementary Material 1: Tables of perioperative and follow-up data [file 12894_2023_1221_MOESM1_ESM.pdf]

Supplementary Table 1 Preoperative baseline characteristics of all patients

**Preoperative characteristics**	**Intervention group**	**Routine RA group**	**TA group**	**OA group**	***p* value**
Gender (n/%)					
Male	16/57.14	15/53.57	16/57.14	14/50.00	> 0.05
Female	12/42.86	13/46.43	12/42.86	14/50.00	
Affected side					
Left	10/35.71	10/35.71	10/35.71	11/39.29	> 0.05
Right	18/64.29	18/64.29	18/64.29	17/60.71	
Tumor maximum diameter (cm)	8.33 ± 1.41	7.98 ± 1.09	8.09 ± 0.75	9.13 ± 2.53	> 0.05
BMI index	22.10 ± 2.15	21.21 ± 1.95	21.62 ± 1.85	22.05 ± 2.06	> 0.05

RA = Retroperitoneoscopic adrenalectomy, TA = Transperitoneal laparoscopic adrenalectomy, OA = Open adrenalectomy.

Supplementary Table 2 Perioperative and follow-up measurements of all patients

Measurements	Intervention group	Routine RA group	TA group	OA group
Intraoperative bleeding volume (ml)[a]	20.00 [10.00-50.00]	50.00 [30.00-82.50]*	55.00 [30.00-300.00]**	300.00 [200.00-600.00]***
Operation time (h)	115.32 ± 30.69	162.96 ± 50.83*	153.04 ± 9.96**	172.18 ± 75.96***
Highest intraoperative systolic pressure (mmHg)	161.96 ± 25.94	188.21 ± 30.80*	187.50 ± 21.02**	198.93 ± 16.63***
Lowest intraoperative systolic pressure (mmHg)	102.86 ± 14.93	95.00 ±14.72	108.39 ± 11.95	97.86 ± 16.63
Range of intraoperative systolic pressure variation (mmHg)	59.11 ± 25.68	93.21 ± 37.40*	79.11 ± 23.88**	101.07 ± 24.99***
Patients transferred to ICU after surgery (n/%)	2/7.14	7/25.00*	9/32.14**	14/50.00***
Time length of postoperative drainage (days)	2.57 ± 0.50	3.21 ± 0.42*	3.82 ± 0.90**	6.82 ± 0.77***
Postoperative diet initiation time (days after surgery)	1.32 ± 0.48	1.43 ± 0.50	2.82 ± 0.39**	2.79 ± 0.42***
Postoperative ambulation time (days after surgery)	2.68 ± 0.48	2.79 ± 0.57	3.18 ± 0.39**	5.21 ± 0.42***

Pain scores	3.21 ± 0.63	3.50 ± 0.51	3.00 ± 0.82	7.04 ± 0.64***
Total length of hospitalization (days)	7.21 ± 1.73	8.14 ± 1.41	8.54 ± 1.60**	11.18 ± 1.06***
Postoperative complications (n/%)				
Postoperative gastrointestinal complaints	2/7.14	5/17.86	16/57.14**	9/32.14***
Hypotension	8/28.57	6/21.43	6/21.43	7/25.00
Hypoglycemia	2/7.14	4/14.29	3/10.71	5/17.86
Delayed wound healing	0/0	0/0	0/0	2/7.14
DVT	1/3.57	2/7.14	1/3.57	3/10.71
Median followed-up systolic pressure (mmHg)	123.68 ± 12.54	128.25 ± 13.26	118.25 ± 9.85	125.04 ± 11.53
Median followed-up diastolic pressure (mmHg)	93.86 ± 8.91	91.12 ± 9.63	90.04 ± 7.93	89.61 ± 7.82
One-month re-check of plasma metanephrine	All in normal range	All in normal range	All in normal range	One patient had a 6-fold elevation[b]

One-month re-check of plasma normetanephrine	All in normal range	All in normal range	All in normal range	One patient had a 2-fold elevation[b]

[a] Non-normally distributed continuous data, which are presented as median (interquartile range [IQR], 25–75%), were analyzed using the Mann-Whitney U test.

[b] One patient in the OA group had poor control of blood pressure, with a 6-fold elevation in metanephrine and 2-fold elevation in normetanephrine. The patient was diagnosed with metastases after resection of the primary tumor.

* Statistically significant differences exist between the routine RA group and intervention group ($p < 0.05$).

** Statistically significant differences exist between routine TA group and intervention group ($p < 0.05$).

*** Statistically significant differences exist between routine OA group and intervention group ($p < 0.05$).

RA = Retroperitoneoscopic adrenalectomy, TA = Transperitoneal laparoscopic adrenalectomy, OA = Open adrenalectomy, DVT = Deep venous thrombosis.
